# Unraveling the epigenetic landscape of pulmonary arterial hypertension: implications for personalized medicine development

**DOI:** 10.1186/s12967-023-04339-5

**Published:** 2023-07-17

**Authors:** Jaydev Dave, Vineeta Jagana, Radoslav Janostiak, Malik Bisserier

**Affiliations:** 1 Department of Cell Biology and Anatomy, New York Medical College, 15 Dana Road, BSB 131A, Valhalla, NY 10595 USA; 2 Department of Physiology, New York Medical College, 15 Dana Road, BSB 131A, Valhalla, NY 10595 USA; 3 First Faculty of Medicine, BIOCEV, Charles University, Vestec, 25250 Czech Republic

**Keywords:** Epigenetic, Pulmonary arterial hypertension, Treatment, Epidrugs, PAH

## Abstract

Pulmonary arterial hypertension (PAH) is a multifactorial disease associated with the remodeling of pulmonary blood vessels. If left unaddressed, PAH can lead to right heart failure and even death. Multiple biological processes, such as smooth muscle proliferation, endothelial dysfunction, inflammation, and resistance to apoptosis, are associated with PAH. Increasing evidence suggests that epigenetic factors play an important role in PAH by regulating the chromatin structure and altering the expression of critical genes. For example, aberrant DNA methylation and histone modifications such as histone acetylation and methylation have been observed in patients with PAH and are linked to vascular remodeling and pulmonary vascular dysfunction. In this review article, we provide a comprehensive overview of the role of key epigenetic targets in PAH pathogenesis, including DNA methyltransferase (DNMT), ten-eleven translocation enzymes (TET), switch-independent 3A (SIN3A), enhancer of zeste homolog 2 (EZH2), histone deacetylase (HDAC), and bromodomain-containing protein 4 (BRD4). Finally, we discuss the potential of multi-omics integration to better understand the molecular signature and profile of PAH patients and how this approach can help identify personalized treatment approaches.

## Introduction

**Fig. 1:**
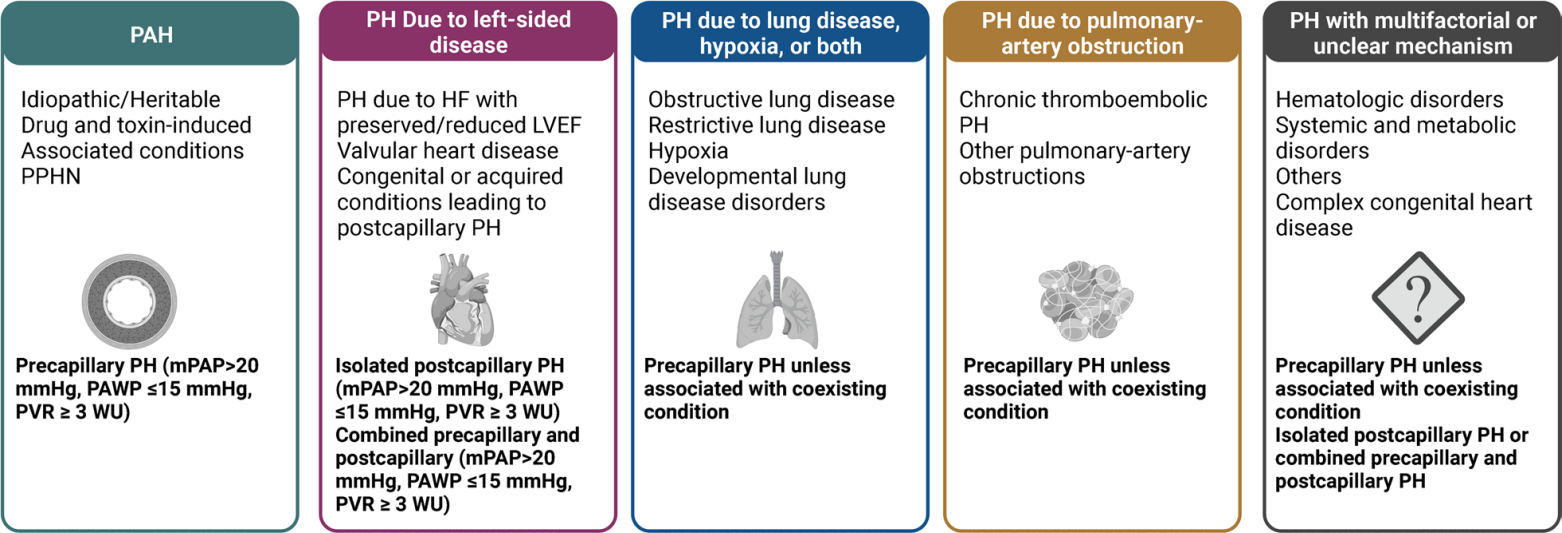
Clinical classification of pulmonary hypertension. The World Health Organization classifies PH into five groups based on its etiology, clinical presentation, hemodynamics, and characteristics. Group 1 includes pulmonary arterial hypertension (PAH), which is idiopathic, heritable, or associated with conditions such as connective tissue disease, congenital heart disease, HIV infection, or portal hypertension. Group 2 includes PH due to left heart disease. This type of PH is caused by left ventricular (LV) dysfunction or valvular disease. Group 3 includes PH due to lung diseases and/or hypoxia, such as chronic obstructive pulmonary disease, interstitial lung disease, and sleep-disordered breathing. Group 4 includes chronic thromboembolic pulmonary hypertension caused by recurrent thromboembolic occlusion of the pulmonary arteries. Group 5 includes PH with unclear and/or multifactorial mechanisms. Classification of PH is crucial for accurate diagnosis, appropriate treatment, and a better understanding of the pathophysiology of the disease. *HF* heart failure, *LVEF* left ventricular ejection fraction, *mPAP* mean pulmonary arterial pressure, *PAH* pulmonary arterial hypertension, *PAWP* pulmonary arterial wedge pressure, *PVR* pulmonary vascular resistance, and *WU* wood units. Created with BioRender.com

Pulmonary arterial hypertension (PAH) is a multifactorial disorder that is associated with severe structural and functional pathological changes in the cardiopulmonary system. PAH is characterized by pulmonary vascular remodeling and a progressive and sustained increase in pulmonary vascular resistance that increases blood pressure in small pulmonary arteries [1, 2]. It is defined hemodynamically as a mean pulmonary arterial pressure > 20 mmHg at rest that increases by more than 3 Wood Units in pulmonary vascular resistance (PVR), as measured by right heart catheterization [1, 2]. The World Health Organization (WHO) categorizes pulmonary hypertension into five subgroups based on its etiology (Fig. 1) [1, 2]. PAH refers to Group 1, a relatively rare form of pulmonary hypertension (PH) and includes idiopathic pulmonary arterial hypertension (IPAH), heritable PAH (HPAH, formerly known as familial or genetic PAH), and other forms of PAH associated with another disease or condition such as scleroderma, schistosomiasis, lupus, congenital heart disease, chronic liver disease, HIV, drugs, and toxins [3]. Group 2 includes PH associated with left-heart diseases, such as heart failure with preserved and reduced left ventricular ejection fraction and congenital and acquired conditions that cause postcapillary PH. Group 3 includes PH due to lung disease and/or hypoxia, such as obstructive and restrictive pulmonary diseases and hypoxia without lung disease and developmental lung disorders. Group 4 PH describes patients with pulmonary artery obstruction caused by blood clots. Group 5 PH is associated with several distinct mechanisms, such as metabolic disorders, hematological disorders, congenital heart disease, cancer-related causes, and systemic disorders, such as sarcoidosis and glycogen storage disease [4]. Recent studies estimate the prevalence of PAH to be between 5 and 15 cases per million individuals in the United States [5, 6]. Epidemiological studies have shown that women have a higher incidence rate than men across multiple studies (4.3:1 in the total PAH group and 4.1:1 in the IPAH category) [7]. The prevalence of PAH in females is significantly higher than the 1.7:1 female-to-male ratio reported by the National Institutes of Health (NIH) registry, which studied only the total population without analyzing subcategories. Based on a study conducted in the 1990s, the NIH reported that the median survival of patients with PAH was 2.8 years without appropriate medical treatment. More recently, the Registry to Evaluate Early and Long-term PAH Disease Management (REVEAL) evaluated patients between 2006 and 2012 and reported a significant improvement in outcomes in the current era [8]. Indeed, data from the REVEAL Registry showed that the median survival rate had improved slightly and now exceeds seven years. This may be partly explained by improvements in therapeutic modalities, patient support strategies, and possibly a PAH population at variance with other cohorts. However, due to the complexity and heterogeneity of factors associated with PAH, the disease is often diagnosed late in its course, usually when the pathological changes are already advanced and irreversible.

## Symptoms and diagnosis

Common symptoms of PAH include non-specific clinical signs such as shortness of breath, chest pain, syncope upon exertion, fatigue, and edema [9]. Vasoconstriction and vascular remodeling are the primary causes of these symptoms. These pathological changes are responsible for the loss of the vascular cross-sectional area, increased pulmonary vascular resistance (PVR), and increased right ventricular afterload, eventually contributing to right ventricle (RV) failure and death [10]. The early diagnosis of PAH is often challenging and almost impossible because of the variety of non-specific symptoms associated with PAH. A thorough investigation is necessary to rule out acute causes. Furthermore, additional underlying medical conditions such as HIV or connective tissue diseases increase the chance of patients developing PH [1]. A physical examination pushing the diagnosis towards PH would include murmur sounds and evidence of right ventricular fluid overload, such as increased jugular venous pressure and peripheral edema.

Diagnostic testing includes procedures such as transthoracic echocardiography (TTE) to evaluate the severity and cause and validate the diagnosis of Group 2 PH. Echocardiography is usually performed to assess right and left ventricular dysfunction, in addition to right heart catheterization, to directly measure the pressure inside the pulmonary artery and right ventricle. Additionally, values related to peak tricuspid regurgitation velocity (TRV) are obtained. The TRV is a crucial part of the algorithm and is used to parse echocardiography data to determine the probability of PH and the necessity for further testing. Cardiac magnetic resonance imaging (MRI) is also recommended to obtain functional information on blood flow, stroke volume, cardiac output, RV size and mass, and the pulmonary vasculature. Cardiac MRI in patients with PAH often shows signs of retrograde blood flow, RV hypertrophy or dilation, reduced distensibility of the pulmonary arteries, or evidence of obstruction or emboli. This information should be combined with other clinical and diagnostic results to diagnose PH accurately [1, 4].

## Pathophysiology of PAH

**Fig. 2:**
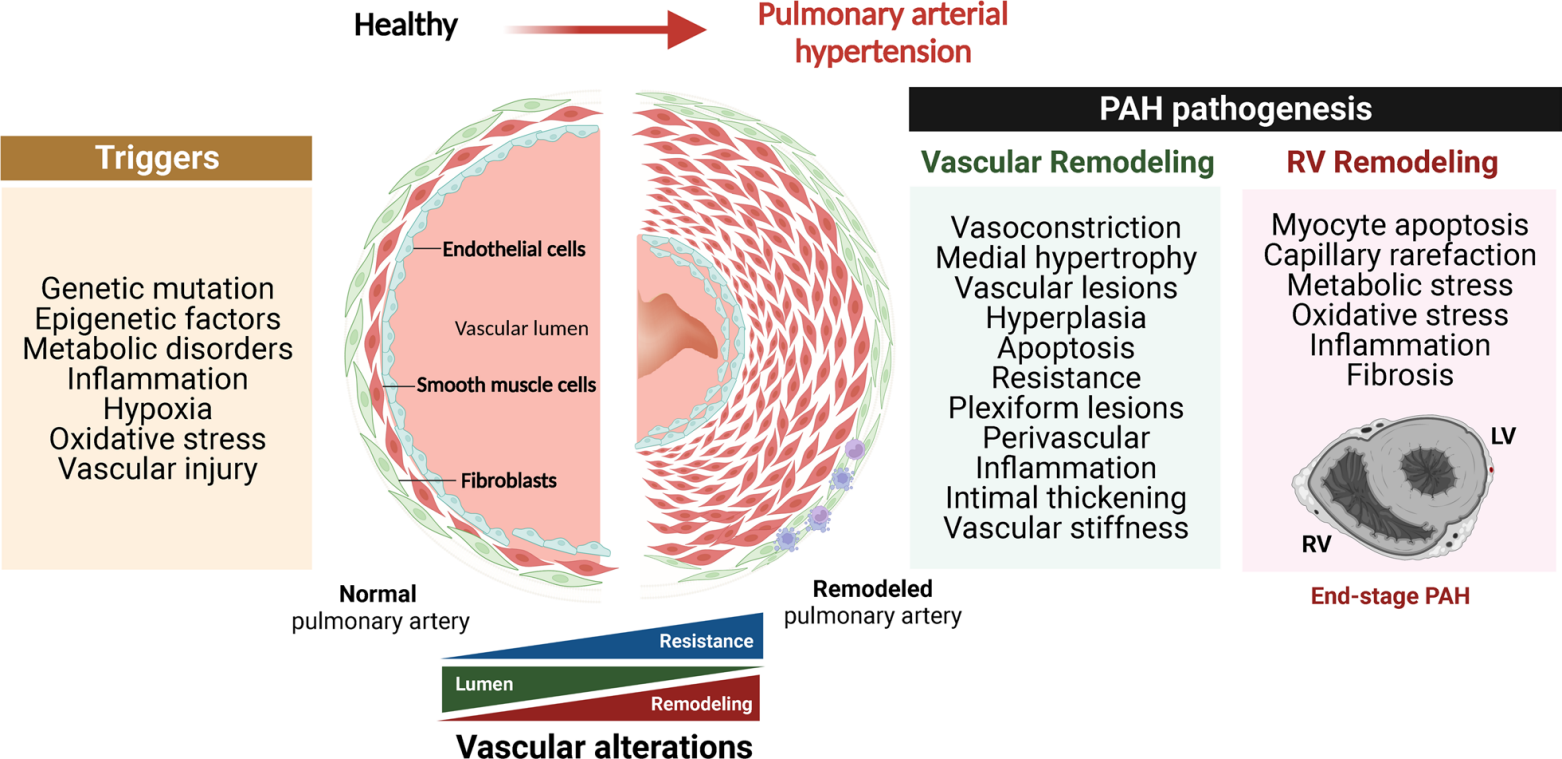
Overview of the pathogenesis of PAH: triggers and cellular and biological alterations. PAH is characterized by the thickening of the pulmonary artery walls and a decrease in lumen size, leading to increased pulmonary vascular resistance and right ventricular failure. Triggers include genetic/epigenetic alterations, inflammation, infections, hypoxia, and drugs/toxins, which promote a pro-inflammatory and prothrombotic phenotype in PAEC, PASMC, and fibroblasts. This leads to vasoconstriction, cell proliferation/migration, and the inhibition of apoptosis. Media thickening also contributes to vessel narrowing. Late-stage PAH is often accompanied by decompensated RV failure, associated with capillary rarefaction, metabolic changes, oxidative stress, inflammation, fibrosis, and neurohormonal activation. *LV *left ventricle, *PAH* pulmonary arterial hypertension, *RV* right ventricle. Created with BioRender.com

The underlying mechanisms that precipitate pathological vascular remodeling stem from the different cell types in the vascular wall (Fig. 2). Dysfunction of pulmonary artery smooth muscle cells (PASMC) and pulmonary artery endothelial cells (PAEC) leads to uncontrolled cell proliferation, thrombosis, medial hypertrophy, migration, metabolic changes, and increased inflammatory responses [11]. Over time, these insults lead to the formation of plexiform lesions causing further disruption of the vascular microenvironment [12, 13]. Endothelial cell dysfunction also impairs the balance between vasodilators and vasoconstrictors, which leads to increased PVR and contractility of the pulmonary arteries, vasoconstriction, and RV hypertrophy (Fig. 2). Together, these factors contribute to RV dysfunction [14].

### Epigenetic dysregulation in PAH

**Fig. 3:**
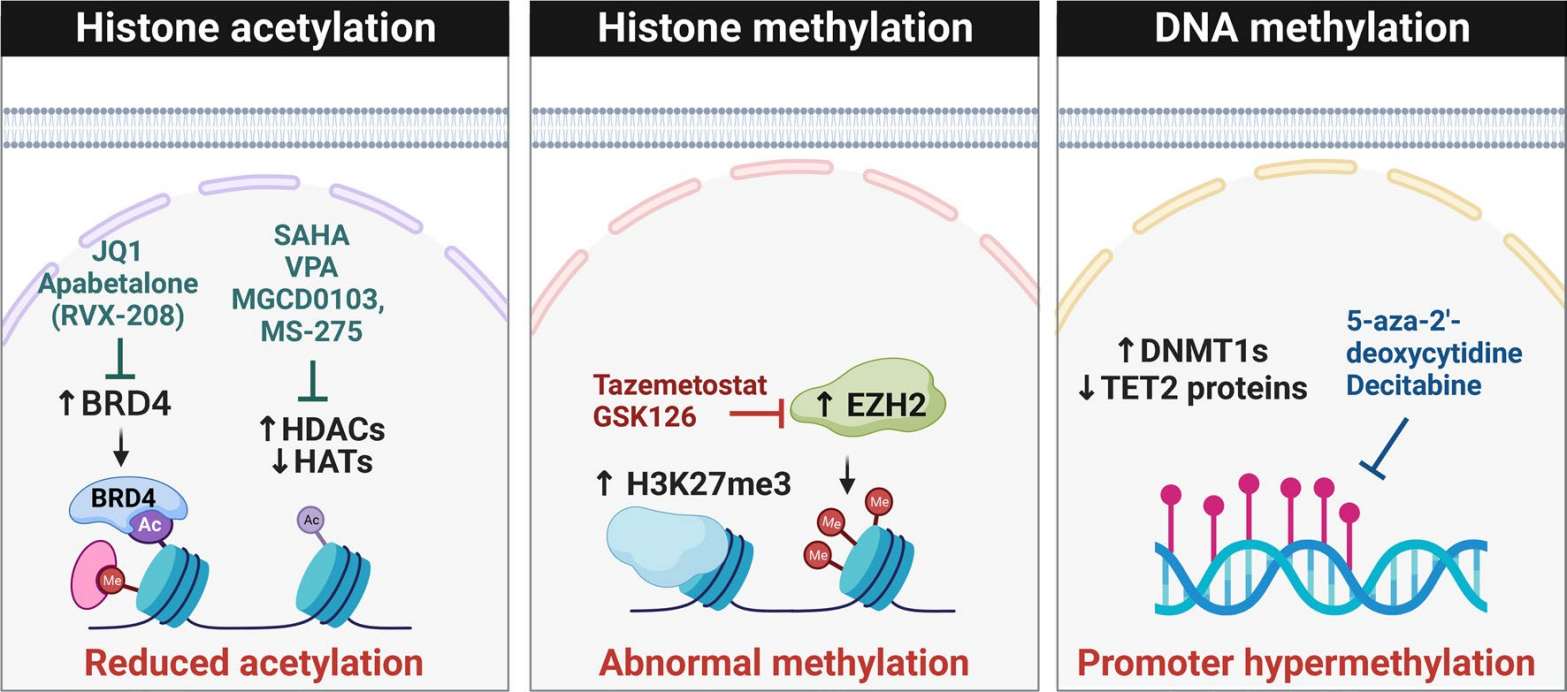
Epigenetic modifications and potential therapeutic targets for PAH. Epigenetic modifications such as DNA methylation, histone acetylation, and methylation play crucial roles in PAH. Increased HDAC activity in PAH represses the expression of genes involved in vasodilation and angiogenesis. EZH2, a histone methyltransferase enzyme that catalyzes the methylation of lysine 27 on histone H3, is also implicated in PAH pathogenesis. Furthermore, the epigenetic reader BRD4 is a transcriptional co-activator that binds to acetylated lysine residues on histone proteins and facilitates recruitment of transcriptional machinery to target genes. Additionally, DNMT1, TET1, and TET2 dysregulation induces aberrant DNA methylation patterns that contribute to the development and progression of PAH. Targeting epigenetic modifications with drugs such as BET (JQ1, apabetalone [RVX-208]), DNMT (5ʹ-aza-2ʹdeoxycytidine, decitabine), HDAC (SAHA, VPA, MGCD0103, MS-275), and EZH2 (tazemetostat, GSK126) inhibitors has shown promise as potential therapies for PAH by reversing the hyperproliferative and anti-apoptotic phenotypes of cells in the pulmonary vasculature. *DNMT* DNA methyltransferase, *TET* ten-eleven translocation methylcytosine dioxygenase, *BRD4* bromodomain-containing protein 4, *EZH2* enhancer of zeste homolog 2, *HDAC* Histone Deacetylase, *H3K27me3* histone 3 lysine 27 trimethylation, *Me* methylation, *Ac* acetylation, *SAHA* suberoylanilide hydroxamic acid, *VPA* valproic acid. Created with BioRender.com

Epigenetics refers to the heritable changes occurring in the genome that result in changes in gene expression without affecting the DNA sequence. These heritable changes occur through specific epigenetic modifications known as epigenetic marks, including DNA methylation, histone modifications, or chromatin-independent epigenetic layer of non-coding RNAs (ncRNAs) (Table 1). They can turn the genes ‘off’ and ‘on’ by directly impacting chromatin packaging, accessibility or mRNA and protein expression. DNA and histone modifications are mediated by three main mediators: writers, erasers, and readers. Writers are directly responsible for modifying histone nucleotides or specific amino acid residues in the DNA. Erasers are responsible for the removal of these marks. Finally, readers refer to all the proteins that contain a specialized domain capable of recognizing specific epigenetic marks. In mammals, increasing evidence over the past decade has shown that epigenetic changes are critical for embryonic development, differentiation, genomic imprinting, X-chromosome inactivation, and repression of tumor suppressors. Aberrant DNA methylation and histone modifications, such as histone acetylation and methylation, have been reported in PAH patients and are linked to vascular remodeling and pulmonary vascular dysfunction (Fig. 3). Additionally, non coding RNAs belong to a separate group of epigenetic modifiers, that have various functions and affect several layers of gene expression, from chromatin structure to RNA turnover or translatability. Similar to epigenetic marks on DNA, ncRNAs are known to play a crucial role in pathogenesis of PAH. The above mentioned epigenetic mechanisms are discussed further in this review article.

**Table 1:** Overview of epigenetic marks, enzymes, biological processes, and signaling pathways in PAH

Type	Epigenetic marks	Enzyme	Transcriptional Activity	Biological processes	Signaling pathway	Therapeutic interventions	References
Histone methylation	H3K27me3	EZH2	Repression	Cell proliferation, migration, apoptosis, autophagy, energy production, survival	SMAD 1/5/9	Tazemetostat, GSK126	[68]
Histone acetylation	H3K27ac	p300	Activation	Cell growth, proliferation, differentiation	BRD4	JQ1 RVX208, Apabetalone	[69–73], [141]
	/	HDAC1, HDAC2 HDAC5, HDAC6	Repression	Cell proliferation, differentiation, and survival	BRD4	VPA, MGCD0103, MS-275, SAHA, Vorinostat, TubA, ACY-775	[54, 55]
Non-coding RNA	microRNA	miR204	Repression	Cell proliferation, migration, and contraction	BMPR2, ALK1, SMAD, NFAT, STAT3, BRD4	Synthetic miR-204 (miR-204 mimic)	[98–101]
		miR17/92	Repression	Proliferation, apoptosis resistance	Mst1, Akt, PDH2, HIF1, VEGF, Glut1, HK2, PDK1	miRVana™ miRNA inhibitors	[92–95]
	lncRNA	lncRNA-MEG3, lncRNA-GAS5, lncRNA-CASC2	Repression	Cell cycle, proliferation, migration, angiogenesis, NO production, oxidative stress.	Cyclin A, Cyclin E, β-catenin, c-Myc, cyclinD1, PPARδ	R8-Lip-siMEG3 GAS5-Contained Exosomes	[106–126]
DNA methylation	5mc; 5hmc	DNMT1 DNMT3a, DNMT3b	Repression	Proliferation, migration, oxidative stress, oxidative stress	SOD2, BMPR2, SMAD1/5/9	5-aza-2′-deoxycytidine Decitabine	[15], [21–28]
		TET1, TET2	Activation	Inflammation, proliferation, apoptosis.	Il1b, Cxcr2, Csf3r, Ccr1, Mmp9, Cd33, Itgam, Il1R, Il1R2	/	[31–33]

This table provides a comprehensive overview of key epigenetic marks, enzymes, biological processes, and signaling pathways associated with pulmonary arterial hypertension (PAH). It covers various epigenetic modifications such as histone methylation, histone acetylation, histone deacetylation, non-coding RNA (including microRNA and lncRNA), and DNA methylation. Additionally, therapeutic interventions targeting these epigenetic processes are provided

*H3K27me3* histone 3 lysine 27 trimethylation, *EZH2* enhancer of zeste homolog 2, *HDAC* histone deacetylase, *p300* E1A binding protein P300, *BMPR2* bone morphogenetic protein receptor 2, *ALK1* activin receptor-like kinase 1, *NFAT* nuclear factor of activated T cells, *STAT3* signal transducer and activator of transcription 3, *BRD4* bromodomain-containing protein 4, *miR* microRNA, *lncRNA* long non-coding RNA, *MEG3* maternally expressed gene 3, *GAS5* growth arrest-specific 5, *CASC2* cancer susceptibility 2, and *PPARδ* peroxisome proliferator-activated receptor δ, *5mc* 5-methylcytosine, *5hmc* 5-hydroxymethylcytosine, *DNMT* DNA methyltransferase, *TET1, TET2* ten-eleven translocation 1, ten-eleven translocation 2, *Il1b* interleukin-1 beta, *Cxcr2* C–X–C motif chemokine receptor 2, *Csf3r* colony stimulating factor 3 receptor, *Ccr1* C–C chemokine receptor 1, *Mmp9* matrix metallopeptidase 9, *Cd33* cluster of differentiation 33, *Itgam* integrin subunit alpha M, *Il1R* interleukin-1 receptor, *Il1R2* interleukin-1 receptor 2

### DNA methylation

DNA methylation is an epigenetic mechanism in which enzymes known as DNA methyltransferases (DNMTs) transfer a methyl group from S-adenyl methionine (SAM) to the fifth carbon of the cytosine residue to form 5-methylcytosine (5-mC) on a DNA molecule [15]. Most DNA methylation occurs on cytosines that precede guanine nucleotides or CpG sites. CpG islands, particularly those associated with promoters, are highly conserved between mice and humans. DNA methylation regulates gene expression by modulating the chromatin structure and disrupting the binding of transcriptional activators. DNA methylation can directly recruit proteins involved in gene repression to regulate gene expression. Other proteins, such as methyl-CpG-binding domain proteins (MBDs), can also recognize DNA methylation and inhibit transcription factor binding [16]. For example, MBD 1–4 and methyl CpG mbinding proteins (MeCPs) such as MeCP 1 and 2 work in a complex with other proteins to condense chromatin and repress gene expression [17, 18]. DNA methylation also suppresses transcription by recruiting DNMTs and lymphocyte-specific helicases (LSH), which can modify chromatin structure [19]. Interestingly, DNA methylation is inherently mutagenic, because methylated cytosine can spontaneously deaminate to thymine. Consequently, mutations acquired over the course of evolution or as a result of environmental risk factors have significantly decreased the number of CpG dinucleotides in the mammalian genome, and mammals have shown lower levels of CpG dinucleotides than expected [20]. As a result, the few remaining CpG sites in the human genome are subject to tight regulation via the activity of DNMTs and Ten-eleven translocation (TET) proteins.

#### DNA methyltransferases (DNMTs)

DNMT1 is a major enzyme involved in DNA methylation inheritance. During DNA replication, DNMT1 interacts with hemi-methylated DNA and copies the DNA methylation pattern from the parental DNA strand to a newly synthesized daughter strand. These are the known maintenance devices. In contrast, Dnmt3a and Dnmt3b are essential for de novo methylation. They preferentially bind to unmethylated DNA and add methyl groups to CpG sites to establish new methylation patterns [21]. DNMT1 exists in an autoinhibitory state by default because of its replication foci targeting sequence (RFTS), which binds to and inhibits the activity of its catalytic domain [22]. To eliminate this inhibition and effectively induce DNA methylation, DNMT1 requires the presence of another protein, UHRF1, an E3 ubiquitin-protein ligase. Briefly, UHRF1 binds to DNMT1 via its ubiquitin-like (UBL) domain, and the UHRF1-DNMT complex is then recruited to the replication fork. UHRF1 binds to hemi-methylated DNA via SET and RING RING-associated (SRA) domains [23]. UHRF1 also has tandem Tudor and PHD (TTD-PHD) domains that recognize and bind histone H3K9me2 [24]. UHRF1 utilizes its RING finger domain to ubiquitinate histone H3 tails, allowing RFTS to bind to the ubiquitinated tails rather than to the DNMT1 catalytic domain. With RFTS outside the catalytic site, DNMT1 becomes catalytically active and can methylate DNA [25]. UHRF1 is an essential component of the methylation machinery, as illustrated by UHRF1 knockout models that replicate the phenotype of DNMT1 knockouts [23].

#### Tet methylcytosine dioxygenase 2

In contrast, TET enzymes mediate the removal of methylation marks and consequent transcriptional activation of a particular gene. TET methylcytosine dioxygenases can oxidize the 5-methylcytosine (5-mc) to 5-hydroxymethylcytosine (5-hmC), 5-formylcytosine (5-fC), and 5-carboxycytosine (5-caC) [26, 27]. These substrates allow for passive and active DNA demethylation mechanisms to occur. Passive demethylation occurs because oxidized 5-mc (5-hmC, 5-fC, and 5-caC) is not recognized by DNMT1. Therefore, DNMT1 does not transfer methylation marker groups to the corresponding cytosine on the nascent strand during replication [28]. As a result, there is a “passive” replication-dependent loss of methylation when DNMT1 comes across an oxidized cytosine. Notably, multiple mechanisms are involved in active demethylation of DNA. The oxidation of 5-mC to 5-hmC by TET is the first enzymatic step that leads to the restoration of unmodified cytosine. For example, one of the proposed mechanisms is that once the methylated cytosine is oxidized to 5-fC or 5-caC by TET proteins, the DNA repair enzyme thymine-DNA-glycosylase (TDG) utilizes the base excision repair pathway to replace the modified base with a naked cytosine, thus demethylating the DNA [29]. Another mechanism of demethylation relies on the deamination-mediated pathways of 5-mC and 5-hmC by the activation-induced cytidine deaminase/apolipoprotein B mRNA-editing enzyme complex (AID/APOBEC) enzymes, which hydroxylate the methyl cytosine to form 5-hmC instead of going through another round of oxidation, and 5-hmC is deaminated [30]. AID/APOBEC basically converts 5-mc into 5-hydroxymethyluracil (5-hmU). This results in a G/T base mismatch that can be fixed via the BER pathway, allowing for the transfer of unmodified cytosine [31].

Dysregulation and dysfunction of TET protein expression and/or activity have been linked to multiple types of cancer pathogenesis and uncontrolled proliferation in a wide range of cancer types, including gastric, prostate, liver, lung, and breast cancers as well as glioblastoma and melanoma [32]. Recently, Potus et al. identified *TET2* as a gene associated with PAH [33]. Using a cohort of 2572 cases from the PAH Biobank, the authors identified numerous deleterious germline and somatic variants of *TET2*. The authors reported that inherited and acquired abnormalities in TET2 occur in 0.39% of PAH cases, with 75% predicted germline mutations and 25% predicted somatic mutations in *TET2* [33]. Using peripheral blood mononuclear cells from an independent cohort of 140 patients, the authors showed that circulating TET2 was significantly downregulated in 86% of PAH patients and was associated with increased inflammation. To further characterize the functional consequences of TET2 depletion in PAH, *Tet2* hematopoietic conditional knockout (KO) mice were generated. *Tet2*-depleted mice spontaneously developed PAH, as evidenced by increased right ventricular systolic pressure, total pulmonary resistance, decreased pulmonary artery acceleration time, adverse pulmonary vascular remodeling, and inflammation [31]. Their results in heterozygous *Tet2*^+/−^ mice revealed that partial loss of *Tet2* is sufficient to induce PAH but is less pronounced, suggesting a gene dose-effect response [33]. Collectively, this study further emphasizes the importance of TET proteins and DNA methylation in PAH pathogenesis.

In 2022, a very interesting study from D’Addario et al. analyzed the differences in the expression of DNMTs and TETs in leukocytes and the severity of pulmonary arterial hypertension between ethnic groups [34]. Their findings indicated that (1) the expression levels of DNMTs (3a and 3b) and TETs (2 and 3) were higher in PAH patients than in healthy controls and (2) there were significant differences in the expression of these epigenetic enzymes based on ethnicity [34]. Specifically, DNMT1 was downregulated in Hispanic/African American patients with scleroderma-associated and idiopathic patients, whereas TET2/TET3 expression was up-regulated compared to that in Caucasians [34]. Because altered DNA methylation and reduced expression of TET enzymes have been shown to elevate inflammatory cytokines and to be associated with hematological disorders or malignancies, the authors further assessed cytokine levels in Caucasian versus Hispanic/African American PAH patients. The authors found higher IL6 and CCL5 expression in Caucasians than in Hispanic/African American patients.

DNA methylation plays a critical role in the vascular pathology of PH, and accumulating evidence has confirmed a link between DNA methylation and vascular remodeling in PAH [11, 35–37]. For example, DNA hypermethylation has been associated with aberrant cell proliferation and resistance to apoptosis in the small pulmonary arteries. In 2010, Archer et al. demonstrated selective hypermethylation of a CpG island in the enhancer region of intron 2 and another in the promoter region of the mitochondrial superoxide dismutase 2 (SOD2) gene using genomic bisulfite sequencing [38]. SOD2 is a major regulator of hydrogen peroxide production in cells, converting the highly reactive superoxide (O2^•–^) generated from oxidative phosphorylation to less reactive H_2_O_2_ [39]. In PAH, SOD2 expression levels are significantly decreased [40]. Thus, limiting the excessive accumulation of superoxide prevents the oxidation of other lipids, DNA, and proteins. In this study, the authors showed that SOD2 downregulation promotes the activation of hypoxia-inducible factor (HIF-1α), a transcription factor that maintains oxygen homeostasis. HIF signaling is activated under hypoxic conditions and can initiate a myriad of adaptive responses such as angiogenesis, proliferation, oxygenation, cellular metabolism, and inflammation [41]. In PAH, low SOD2 expression increases ROS production and activates HIF-1α signaling, potentiating the expression of oxygen-sensitive voltage-gated K + channels (Kv1.5) [38]. Together, these physiological responses cause defects in oxygen sensing and reduce the cytoplasmic and mitochondrial redox states. The authors found that hypermethylation in the promoter and enhancer regions of SOD2 repressed SOD2 expression in samples from PAH patients and Fawn-Hooded rats (FHR) [38]. In line with increased methylation levels, they also found that DNMT1 and DNMT3B were overexpressed in FHR lungs, which caused them to spontaneously develop pulmonary hypertension. Thus, SOD2 dysregulation contributes to the apoptosis-resistant and hyperproliferative phenotypes in PASMC. Mechanistically, this study demonstrated that the pharmacological inhibition of DNMT using the DNA methyltransferase inhibitor 5-aza-2′-deoxycytidine restored both SOD2 expression and the proliferation/apoptosis ratio.

More recently, another study by the same group investigated the role of epigenetic metabolic reprogramming in right ventricular fibroblasts in PAH [42]. In fact, this is the first study demonstrating that epigenetic alterations impair mitochondrial redox signaling by impairing SOD2-mediated production of H_2_O_2_, creating a state of pseudo-hypoxia that leads to normoxic HIF-1α activation in monocrotaline (MCT)-derived right ventricular fibroblast (RVfib) [42]. Their results revealed an excess of mitochondrial fission and aberrant levels in pyruvate dehydrogenase kinase (PDK) isoforms 1 and 3 in MCT-RVfib and increased PDK1 in RVfib in patients with PAH and RV failure [42]. In line with their previous study, the authors found that DNMT1 upregulation depresses SOD2 expression, reduces mitochondrial H_2_O_2_ production, and triggers normoxic HIF-1α activation in MCT-RVfib. PDK1 inhibits pyruvate dehydrogenase (PDH) activity and is, therefore, responsible for the metabolic shift, also known as the Warburg effect. Finally, the authors showed that HIF-1α promoted the Warburg shift and increased the production of inflammatory cytokines such as connective tissue growth factor (CTGF) and transforming growth factor beta (TGF-β1).

#### SIN3 transcription regulator family member A

Accumulating evidence suggests that DNA methylation is involved in the regulation of bone morphogenetic protein receptor type 2 (BMPR2), a member of the TGF-β superfamily [43–45]. To date, autosomal dominant mutations causing haploinsufficiency or loss of BMPR2 function account for 70% of familial PAH (FPAH) cases and 20% of sporadic cases of idiopathic PAH [46]. Loss of BMPR2 expression has been observed in the lung vasculature and blood outgrowth endothelial cells of patients with IPAH and HPAH [47]. In 2017, Liu et al. revealed hypermethylation in the promoter region of BMPR2 in familial PAH patients, suggesting that BMPR2 expression may be epigenetically suppressed in PAH [45]. However, the molecular mechanisms implicated in the epigenetic regulation of BMPR2 in the lungs of patients with non-hereditary PAH have been poorly investigated. New insights into the regulation of BMPR2 were recently obtained by Bisserier et al. in 2021 [44]. In this study, the authors investigated the role of switch-independent 3a (SIN3a) in the epigenetic regulation of BMPR2 in PAH.

SIN3a is a master transcriptional scaffold protein and co-repressor that plays an essential role in the regulation of gene transcription and maintenance of chromatin structure in a context-specific manner, and its inappropriate recruitment has been associated with aberrant gene regulation in cancer and cardiovascular diseases [48]. Increasing evidence suggests that SIN3a acts as a transcriptional repressor or activator under specific conditions. The authors also found that SIN3a was significantly downregulated in lung samples from patients with PAH and in animal models of PAH [44]. SIN3a overexpression in PASMCs increases BMPR2 levels and inhibits cell proliferation in vitro. At the molecular level, their data showed that SIN3a modulated BMPR2 expression by silencing the expression of the methyltransferase EZH2 (enhancer of zeste homolog 2), a catalytic subunit of PRC2 (polycomb repressive complex 2) involved in the tri-methylation of lysine 27 on histone H3 (H3K27me3), a repressive chromatin mark [44]. Increased H3K27me3 levels were associated with gene repression.

In vitro, SIN3a overexpression significantly decreased H3K27me3 content in the BMPR2 promoter region, which was consistent with the high BMPR2 expression in PASMC [44]. Moreover, the authors found that SIN3a potentiated DNA demethylation at the BMPR2 promoter region via TET1. Pharmacological inhibition of EZH2 by GSK126 in PASMC potentiates BMPR2 expression and further enhances the effects of SIN3a. Collectively, these results indicated that SIN3a plays a critical role in the regulation of BMPR2 expression by modulating EZH2 and TET1 expression. To further demonstrate the therapeutic potential of these findings, they performed intratracheal delivery of an adeno-associated virus serotype 1 encoding human SIN3a (AAV1.*hSIN3a*) in two distinct rodent models of PAH, MCT rats and Sugen/Hypoxia-mice. The authors observed a significant decrease in DNA methylation of the BMPR2 promoter, which was associated with increased BMPR2 expression in the lungs. Furthermore, lung-targeted delivery of AAV1.*hSIN3a* significantly attenuated vascular and cardiac remodeling as well as several hemodynamic parameters such as decreases in RV systolic pressure and pulmonary artery pressure [44].

### Histone modifications

Histone modification refers to chemical changes that occur in histone proteins that are found in chromatin and play a crucial role in the regulation of gene expression [49]. There are various types of histone modifications including acetylation, methylation, phosphorylation, and ubiquitination [49]. These modifications can alter the structure of chromatin, making it more or less accessible to transcription factors thereby influencing gene expression. It is considered a key mechanism for regulating gene expression and is implicated in various diseases such as cancer, cardiovascular disease, and neurological disorders [50]. Understanding the mechanisms and effects of histone modifications is an active area of research with potential implications for the development of novel therapeutics that target epigenetic processes. Furthermore, histone modifications have been shown to be heritable through cell division and across generations, making them an important area of study in developmental biology and transgenerational inheritance [51]. In the context of PAH pathogenesis, histone modification is an emerging area of research and has been shown to impair the expression of critical genes involved in cell proliferation, apoptosis, migration, and mitochondrial metabolism [52, 53]. Studies have shown that histone deacetylase inhibitors can improve pulmonary hypertension [54–56].

#### Histone deacetylases (HDACs)

Previous studies have identified a significant increase in HDAC1 and HDAC5 protein expression in human lung tissue from IPAH patients as well as lungs and RV from hypoxia-induced PH animal models [54]. Furthermore, research has revealed elevated histone acetylation of H3 and H4 in the promoter area of the eNOS gene in PAEC from infants suffering from Persistent PH of the Newborn (PPHN) [57]. Recent studies have emphasized the critical role of HDACs in RV failure across various experimental models of PH, suggesting that targeting histone acetylation may represent a promising area of research for better understanding the pathogenesis of PAH and developing novel therapeutic strategies. Cho et al. evaluated the therapeutic potential of Valproic Acid (VPA) in rat models of PAH induced by monocrotaline (MCT) and pulmonary artery banding (PAB) [58]. Administration of VPA through drinking water significantly increased histone acetylation in the right ventricle of rats and reduced RV hypertrophy, with improved RV systolic function in MCT-treated animals by echocardiography analysis [58]. A study conducted in 2015 showed that the use of MC1568, a pharmacological inhibitor of class IIa HDACs administered daily via intraperitoneal injection at a dose of 50 mg/kg, restored MEF2 activity in PAECs and attenuated cell migration and proliferation in experimental PAH models [59]. These findings further suggest that HDACs can be considered potential therapeutic targets for treating vascular remodeling diseases.

Bogaard et al. conducted a study investigated the effect of TSA, a broad-spectrum HDAC inhibitor, on RV function and remodeling in rats with PAB-induced PH [60]. Their findings were controversial, as they discovered that, rather than preventing RV remodeling, HDAC inhibition with TSA worsened RV fibrosis and led to increased dysfunction. Importantly, their data suggests that selective HDAC inhibition may be more beneficial than the effects of non-selective pan-HDAC inhibitors, such as TSA. Their work highlighted how the development of more selective HDAC inhibitors that target specific HDAC isoforms may lead to more effective and targeted therapies for treating PAH. This was later supported by Cavasin et al.‘s research on highly selective class I HDAC compounds (HDAC1-3), MGCD0103, and MS-275, using the hypoxia-induced PH rat model [55]. Over the course of 3 weeks, intraperitoneal injections of MGCD0103 (10 mg/kg) and MS-275 (3 mg/kg) reduced class I HDAC catalytic activity in both the RV and the lungs [55]. The study showed that HDAC inhibitors were effective in impairing hypoxia-induced PH and improving various hemodynamics such as RV cardiac output, pulmonary vascular resistance, PA acceleration time (PAAT), velocity time integral (VTI), RV end-systolic pressure, and pulmonary artery pressure. Additional research conducted by Zhao et al. revealed that VPA, along with Suberoyl Anilide Hydroxamic Acid (SAHA), a Vorinostat that inhibits classes I to IV HDAC, successfully reversed hypoxia-induced PH in rats and decreased inflammation by limiting PASMC proliferation [54]. Despite conflicting views regarding the role of HDACs in treating PH, recent research has shown that developing more selective HDAC inhibitors that target specific isoforms can lead to effective and targeted therapies for PAH and related vascular remodeling diseases.

The complexity of RV remodeling in PH is partly explained by the heterogeneity and severity of physiological adaptations, which can be classified as adaptive, maladaptive, compensated, or decompensated [61]. The inhibition of RV response using broad-spectrum HDAC inhibitors may result in adverse outcomes for maladaptive but beneficial outcomes for adaptive hypertrophy. In patients with PAH, it is crucial to determine the type of RV function and remodeling before using general HDAC inhibitors because they may lead to issues such as thrombocytopenia, pulmonary embolism, and electrocardiographic abnormalities in patients with lymphoma. Kim et al. demonstrated that selective inhibition of HDAC IIa may be a more viable option, with fewer unintended effects on other genes, which can help prevent RV dilation [59]. However, there is controversy surrounding the therapeutic application of HDAC inhibitors, which may be, in part, due to differences in PAH model types (Sugen-Hypoxia, MCT, and PAB), treatment protocols (including duration and frequency of use), and selectivity levels among different types of HDAC inhibitors.

Unlike nuclear HDACs, which regulate transcription through epigenetic mechanisms, HDAC6 is exclusively found in the cytoplasm and deacetylates proteins other than histones. Research has shown that HDAC6 overexpression has been linked to various cancers, including hepatocellular carcinoma, glioblastoma, and prostate cancer. A selective inhibitor targeting the domains of HDAC6 has been effective in inhibiting cell growth and cancer progression while also increasing the sensitivity of transformed cells to anticancer agents [62]. Boucherat et al. reported increased expression levels of HDAC6 within the lungs and distal PAs in both PAH patients and animal models induced by MCT or Sugen/hypoxia (Su/Hx) experiments [63]. Compared with control cells, HDAC6 was overexpressed only at the protein level in PAH-PASMCs, PAH-PAECs, and RV from both Su/Hx and MCT rats and patients with PAH. When treated with Tubastatin A (TubA), ACY-775, or siRNA against HDAC6, a significant reduction in the proliferation, migration, and resistance to apoptosis of PAH-PASMCs was observed [63]. Pharmacological inhibition of HDAC6 attenuates pulmonary hypertension by decreasing total pulmonary vascular resistance (TPR), cardiac output (CO), mean PA pressure (mPAP), and RV systolic pressure (RVSP) [63]. This was associated with the restoration of the balance between proliferation and apoptosis in small pulmonary arteries and reduced vascular remodeling and RV hypertrophy in the Sugen/Hypoxia and MCT rat models. These findings suggest that HDAC6 may serve as a promising therapeutic target for PAH. However, further studies are needed to evaluate the selectivity of HDAC6 inhibitors in PAH and to determine their safety for cardiac function.

#### Enhancer of zeste homolog 2 (EZH2)

Overexpression and hyperactivation of enhancer of zeste homolog 2 (EZH2) have been implicated in several cancers, including lung cancer, breast cancer, prostate cancer, and hematological malignancies [64]. Gain-of-function mutations and/or mutations in the catalytic SET domain of EZH2 have been identified in non-Hodgkin lymphoma (NHL), melanoma, and other cancers [64]. EZH2 is a histone methyltransferase that catalyzes the trimethylation of lysine 27 on histone H3, resulting in gene silencing. EZH2 regulates many cellular processes such as cell proliferation, differentiation, and apoptosis. EZH2 inhibitors, such as Tazemetostat (EPZ-6438), GSK126, and CPI-1205, have been developed to treat EZH2 overexpressing cancers and non-oncological diseases [65, 66]. Several EZH2 inhibitors are currently undergoing clinical trials for the treatment of cancers [67]. In addition to cancer, EZH2 plays a role in the pathogenesis of several non-oncologic diseases, including PAH.

Recently, Habbout et al. found that EZH2 is overexpressed in PASMCs from patients with PAH and that EZH2 inhibition with GSK126, an EZH2-specific inhibitor, reduces PASMC proliferation in vitro [68]. This study further demonstrated that EZH2 enhanced the growth and viability of PAH-PASMCs using a gain-of-function approachs. Using quantitative transcriptomic and proteomic techniques, the investigators discovered that suppressing EZH2 significantly reduced the expression of various cell-cycle-related factors, such as E2F targets, while preserving energy production [68]. Their results showed that EZH2 stimulates the transcription of multiple nuclear-encoded elements associated with mitochondrial translation machinery and tricarboxylic acid cycle genes. These findings suggest that EZH2 inhibition reduces PASMC proliferation and enhances mitochondrial function in PAH patients [68]. Additional studies are needed to identify all genes that are regulated by H3K27me3 in the context of PAH to further elucidate the mechanisms underlying EZH2-mediated regulation in PAH. Overall, this study suggests that EZH2 is a potential therapeutic target for the treatment of PAH.

#### Bromodomain-containing protein 4 (BRD4)

Recent studies have shown that the protein BRD4 (Bromodomain-containing protein 4) plays a crucial role in PAH development, making it an important target for potential therapies [69, 70]. BRD4 is a member of the bromodomain and extra-terminal domain (BET) protein family. This family of proteins helps regulate gene expression by binding to acetylated histones, which are involved in DNA organization and packaging. By binding to these acetylated histones, BRD4 plays a role in the formation of transcriptional complexes necessary for the transcription of DNA into RNA [71]. BRD4 also plays a role in regulating the cell cycle, and abnormal expression of BRD4 has been associated with various types of cancers and other diseases [71]. Previous studies have reported that BRD4 activates several downstream targets including the MAPK/ERK pathway, which regulates cell growth and differentiation. Additionally, BRD4 has been shown to interact with other proteins and transcription factors involved in cell proliferation, such as STAT3 [72].

In 2015, Meloche et al. found that BRD4 expression is increased in patients with PAH [69]. The authors found that targeting BRD4 with a small-molecule inhibitor, JQ1, reduced pulmonary vascular remodeling and improved the overall cardiac function in a rat model of PAH [69]. In vitro, JQ1 was found to inhibit the proliferation of human PASMC and improve apoptosis by regulating the expression of several genes involved in cell proliferation, inflammation, and survival pathways, such as the nuclear factor of activated T cells, B-cell lymphoma 2, and Survivin. Mechanistically, the authors also revealed that micro-RNA 204 in PAH is associated with increased BRD4 expression [69]. This study was the first to identify BRD4 as a therapeutic candidate for treating PAH and suggested that targeting BRD4 with specific inhibitors could reduce PASMC proliferation and decrease pulmonary artery pressure, potentially halting or slowing the progression of PAH. Importantly, the identification of BRD4 as a crucial player in PAH development represents an important step forward. BRD4 inhibitors improve pulmonary artery remodeling, reduce right ventricular hypertrophy, and decrease pulmonary vascular resistance in vivo [69]. In 2019, a multicenter study was conducted in three independent laboratories to further validate the therapeutic effect of RVX208, a clinically available BET inhibitor, for treating PAH in several animal models [73]. Their results further confirmed that the remodeled pulmonary vasculature of PAH patients exhibited increased BRD4 expression. This increased expression altered the levels of two major factors in the DNA damage response, FoxM1 and PLK1. By regulating these factors, RVX208 restores normal functionality to microvascular endothelial cells and smooth muscle cells, which are hyperproliferative, apoptosis-resistant, and inflamed [73]. Additionally, in Sugen5416 + hypoxia-PAH and monocrotaline + shunt-PAH models, orally administered RVX208 reversed vascular remodeling and improved pulmonary hemodynamics [73]. Furthermore, it was demonstrated that RVX208 was safe for use along with standard contemporary PAH treatments, and its administration aided pressure-loaded RV in rats undergoing pulmonary artery banding [73]. These promising preclinical results suggest that BRD4 inhibitors such as RVX208 could potentially serve as a novel therapeutic strategy for treating PAH in humans. However, further research is needed to improve our understanding of the efficacy and safety of BRD4 inhibitors.

Recent clinical trials have shown that BRD4 inhibitors may also have a favorable safety profile in humans, making them an attractive target for further development as a treatment option for PAH. The APPRoAcH-p study [NCT03655704] is currently underway to evaluate the safety and efficacy of Apabetalone, a BET inhibitor, in the treatment of PAH. This study enrolled 24 PAH patients who received Apabetalone for 12 weeks. The primary outcome measure was the change in pulmonary vascular resistance, while secondary measures included changes in the 6-minute walk distance, hemodynamic parameters, and biomarkers of PAH. In 2021, Resverlogix announced that the APPRoAcH-p study of Apabetalone met its primary endpoint, demonstrating a statistically significant reduction in pulmonary vascular resistance compared to the baseline. These results provide encouraging evidence for the potential of BET inhibitors such as Apabetalone as promising therapeutic options for treating PAH in humans. In conclusion, the role of BRD4 in PAH provides a new avenue for potential therapies. Although more research is needed to fully understand the efficacy and safety of BRD4 inhibitors in treating PAH and to identify the optimal dosing and treatment duration, promising preclinical and clinical trial results suggest that BRD4 inhibitors could potentially serve as novel and effective treatment strategies for PAH.

### Non-coding RNA

In addition to the well-established epigenetic mechanisms implicated in the development of PAH described in the previous chapters, non-coding RNA has been recognized as an important regulatory mechanism in PAH. Several types of non-coding RNA have been identified and are classified into two major groups based on length. Small non-coding RNAs (e.g. miRNA, snRNA) are generally shorter than 200nt (nucleotides), and long non-coding RNAs (lncRNA, circRNA) are longer than 200nt [74]. The two most described families with functional consequences in PAH are micro-RNA (miRNAs) and long non-coding RNAs (lncRNAs), which will be described in greater detail.

### Micro RNA

miRNAs are short non-coding RNAs with lengths of 21–25 nucleotides. The functional network of individual miRNAs is very broad. For example, one miRNA can regulate the expression of several genes and vice versa [75]. miRNAs are transcribed as pre-miRNAs by RNA polymerase II from intronic or exonic regions as several hundred base-long transcripts containing a 5-m7G cap and 3-poly-A tail [76]. After transcription, they are processed by Drosha in the nucleus, exported to the cytoplasm, spliced by DICER, and loaded into the RNA-induced silencing complex (RISC) [77, 78]. It has been shown that miRNAs regulate all the critical signaling pathways involved in the pathogenesis of PAH [79].

Several key signaling pathways and hubs are altered in patients with PAH, such as the HIF1-signaling axis, BMPR2-TGFβ, PPARγ-signaling axis, and NF-κB-signaling [80]. Previous studies showed that mutations or impairments of these pathways are crucial for PAH development and pathogenicity [80]. MicroRNAs can form positive and/or negative feedback loops with key signaling proteins and transcription factors that contribute to the development of PAH [81, 82].

The key signaling pathway regulating the development of PAH is the BMPR2/ALK1 signaling pathway, and it has been shown that 80% of familial PAH and up to 20% of idiopathic PAH are caused by genetic alterations of this pathway [83]. Multiple miRNAs that affect the levels of several signaling proteins within the BMP/SMAD signaling pathway have been identified. For example, *miR-17-5p* and *miR-20a* repress BMPR2 expression, is regulated by the IL-6/STAT3 signaling axis and is associated with PAH pathology [84]. Similarly, *miR-23a-3p* induced by hypoxia downregulates BMPR2 expression in PASMCs, leading to increased PASMC proliferation and PAH development [85].

ALK1, which inhibits BMPR2, is a target of *miR-98-5p. miR-98-5p* is downregulated in PASMC after hypoxia, which leads to the upregulation of ALK1 and inhibition of BMPR2 signaling [86]. Several miRNAs target the downstream signaling of BMPR2, such as the SMAD proteins. Previous studies showed that miR-199-5p is up-regulated in PAH and is downregulated SMAD3. Similarly, *miR-195-5p* targets SMAD7, promoting PASMC survival [87, 88]. Finally, *miR- 140-5p* downregulates SMAD-specific E3 ubiquitin-protein ligase 1 (SMURF1), leading to stimulation of BMP/SMAD signaling and inhibition of PASMCs proliferation [89]. Interestingly, R-SMADs (SMAD1/5) that are up-regulated through TGF-β1 signaling can stimulate miRNA maturation by binding pre-miRNAs and recruiting Drosha, leading to increased levels of several miRNAs important for PAH, such as *miR-199a* or *miR-21* [90].

Hypoxia plays an important role in the pathogenesis of PAH by inducing oxidative stress. Hypoxia leads to stabilization of the HIF-1α transcription factor, which in turn up-regulates multiple *miRNAs (miR-1, -9, -17, 92, -21, -27a, -27b, -138, -190; -199-5p; -210, -322, -361-5p*, and *-23a)* [11]. HIF-1 stimulates the expression of several miRNAs that directly affect BMP/SMAD signaling (*miR-17-5p, miR-191-5p*, or *miR-195-5p*) [84, 87, 91]. These miRNAs directly increase the survival and proliferation of endothelial and smooth muscle cells in the distal pulmonary arteries, potentiating the development of PAH. Another key miRNA with pleiotropic effects is the miR-17/92 cluster [92]. In addition to the downregulation of BMPR2 and PHD2, the miR-17/92 cluster targets CDKN1A/p21, MFN2, or PTEN [92–94]. miR-138 expression is also regulated by HIF-1α and exerts its function mainly through the regulation of Mst1 and Akt kinases, which promotes apoptosis resistance of PASMC [95].

Finally, another crucial transcription factor implicated in PAH is PPARγ, which negatively regulates PAH development. Previous studies showed that activation of PPARγ counteracts the development of PAH in response to hypoxia by inhibiting HIF-1α [96]. PPARγ is targeted by multiple miRNAs such as miR-*130a-3p, miR-130b-3p, miR-301a-3p, miR-301b-3p, miR-27b-3p, and miR-27a-3p* [81]. Interestingly, miR-27a-3p is up-regulated in response to hypoxia and vice versa, while PPARγ inhibits the production of miR-27a-3p, thus forming a negative feedback loop [97, 98]. A key miRNA cluster that regulates PPARγ is *miR-130/301*, which is up-regulated under hypoxic conditions through the HIF-2α/POU5F1 transcription module [99]. Downregulation of PPARγ, in turn, leads to altered expression of several miRNAs, such as *miR-204-5p, miR-21-5p, miR-27a-3p, and miR-98-5p* [98–101]. Decreased levels of PPARγ result in increased levels of miR-21-5p, which downregulate a broad plethora of targets such as BMPR2, RhoB, PDCD4, SPRY2, PPARα, PTEN, and SATB1, leading to the stimulation of PASMCs and PAECs proliferation, migration, and contraction and ultimately the development of PAH [100, 102, 103]. Additionally, recent reports have shown that FGF21 can increase the expression of PPARγ by downregulating *miR-27a-3p* and *miR-130* and ameliorating hypoxia-mediated PAH development [104, 105].

### Long non-coding RNA

Long non-coding RNAs of more than 200 nucleotides possess various functions based on the sequence and have been implicated in the development of PAH [106, 107]. Like miRNAs, lncRNAs are transcribed by RNA polymerase II or III and processed by the RNA-processing machinery: spliced, 3′-polyadenylated, or 5′-capped [107]. LncRNAs possess various functions: signal lncRNAs regulate the transcription of other genes, decoy lncRNAs sequester regulatory proteins or miRNAs, regulate genes, guide lncRNAs to recruit transcription activators or repressors to specific genome locations, scaffold lncRNAs to form platforms for the assembly of different RNP complexes that control chromatin architecture, and the most recently discovered class are circular lncRNAs that act as transcriptional regulators [108].

The best-studied lncRNA in the context of pulmonary hypertension is *the H19* lncRNA. *H19* is up-regulated in PASMCs in response to IL-1β and PDGF-BB and binds and sequesters let-7b miRNA, leading to the upregulation of AT_1_R expression. AT_1_R stimulates PASMCs proliferation and PAH.

development [109]. In contrast, treatment of PASMC with FGF21 also increased *H19* lncRNA; however, in a study by Li et al., increased *H19* disrupted the mTOR/ EIF4EBP1 complex, which alleviated pulmonary hypertension [110]. Despite these discrepancies, *H19* has been postulated as a marker and therapeutic target in PAH [111]. *MALAT1* is induced by hypoxia and promotes pulmonary vascular remodeling by promoting PSMC proliferation through the regulation of several miRNAs, such as *miR‑124‑3p.1* or *miR-503* [112, 113]. Hypoxia increases the expression of *PAXIP1-AS1* and altered focal adhesion signaling and contractility by up-regulating the paxillin and Rho signaling pathways [114, 115]. Similarly, hypoxia-induced *UCA1* sequesters ING5 from hnRNP I, which leads to PASMC proliferation and resistance to apoptosis [116]. *TUG1* lncRNA is up-regulated by hypoxia in PASMC and pericytes and promotes the proliferation of these cells by regulating other miRNAs, including *miR-328, miR-145-5p, miR-129-5p*, and *miR-138-5p* [117, 118]. Finally, *HOXA-AS3* lncRNA promoted the proliferation and migration of PASMC through at least two identified mechanisms. *HOXA-AS3* stimulates the expression of HoxA3, which results in increased expression of cyclins A, E, and D and increased expression of PDE5A through downregulation of *miR-675-3p* [119, 120]. HOXA-AS3 is a member of the HOX gene clusters and plays a crucial role in embryological development. They are involved in the regulation of hematopoietic lineage and differentiation. Zhu et al. reported that HOXA-AS3 interacts with EZH2 to influence the lineage commitment of mesenchymal stem cells. In the context of glioma, the upregulation of HOXA-AS3 is also associated with tumor progression and is indicative of a poor prognosis. Other components of the HOXA cluster, including HOTAIR and HOTTIP, have been identified as critical in the regulation of cell proliferation in lung cancer.

In contrast, several lncRNAs are downregulated in patients with PAH. Hypoxia downregulates *MEG3* and promotes the progression of the PASMC cycle through several mechanisms, such as the induction of PCNA, Cyclin A, and Cyclin E [121]. Moreover, decreased *MEG3* expression up-regulates *miR-21* and blocks PTEN expression [122]. PAH-associated signaling events, such as hypoxia or increased PDGF-BB levels, decreased the expression of *GAS5* and *CASC2* lncRNAs. Downregulation of *GAS5* promoted the survival and proliferation of PASMC by increasing the expression of *miR-382-3p* and KCNK3 [123, 124]. Similarly, CASC2 downregulation leads to increased miR-222 activity, downregulation of ING5 expression, and stimulation of survival, proliferation, and phenotype switching of PASMCs [125, 126].

## The potential of epidrugs in PAH

Epigenetics can integrate various factors that influence an individual’s health and disease risk, including lifestyle, the environment, and exposure. Environmental factors, including diet, exercise, stress, pollution, and exposure to chemicals and toxins can influence epigenetic modifications [127]. These modifications, in turn, can have long-lasting effects by altering gene expression, affecting an individual’s health and disease risk, and, importantly, being passed on to future generations. For example, maternal diet and exposure to toxins during pregnancy can have epigenetic effects on the developing fetus, influencing the risk of developing various diseases later in life [128]. Given the critical role of epigenetics in the development of PAH, epigenetic drugs, or “epidrugs,“ represent a promising area of research for PAH treatment. Ongoing efforts are exploring the therapeutic potential of new epigenetic modulators, including SIN3a, BRD4, HDACs, TET, and DNMTs, with promising results in preclinical studies. Interestingly, epidrugs may be used in combination with other treatments, such as vasodilators and immune modulators, to improve the outcomes for patients with PAH. Furthermore, as our understanding of the genetic and epigenetic factors underlying PAH improves, epidrugs may be used in a more personalized manner to target specific epigenetic changes unique to each patient. Although the future of epidrugs in the treatment of PAH is encouraging, there is still much to be learned regarding their safety and off-target genes.

## From epigenetics toward multi-omics integration

PAH is a complex disease with multiple contributing factors, including genetic predisposition, environmental exposure, and lifestyle factors, involving multiple biological processes and pathways [76–84], making it challenging to study, diagnose, and treat. Even with genetic sequencing of mutations confirming the diagnosis of heritable PAH, these tests are unique and extremely specific. Furthermore, they are only relevant to a few patients with PAH. Using multi-omics in the context of PAH to identify new therapeutic targets, confirm a diagnosis, or help match patients to the most effective therapy represents a promising approach [129].

**Fig. 4:**
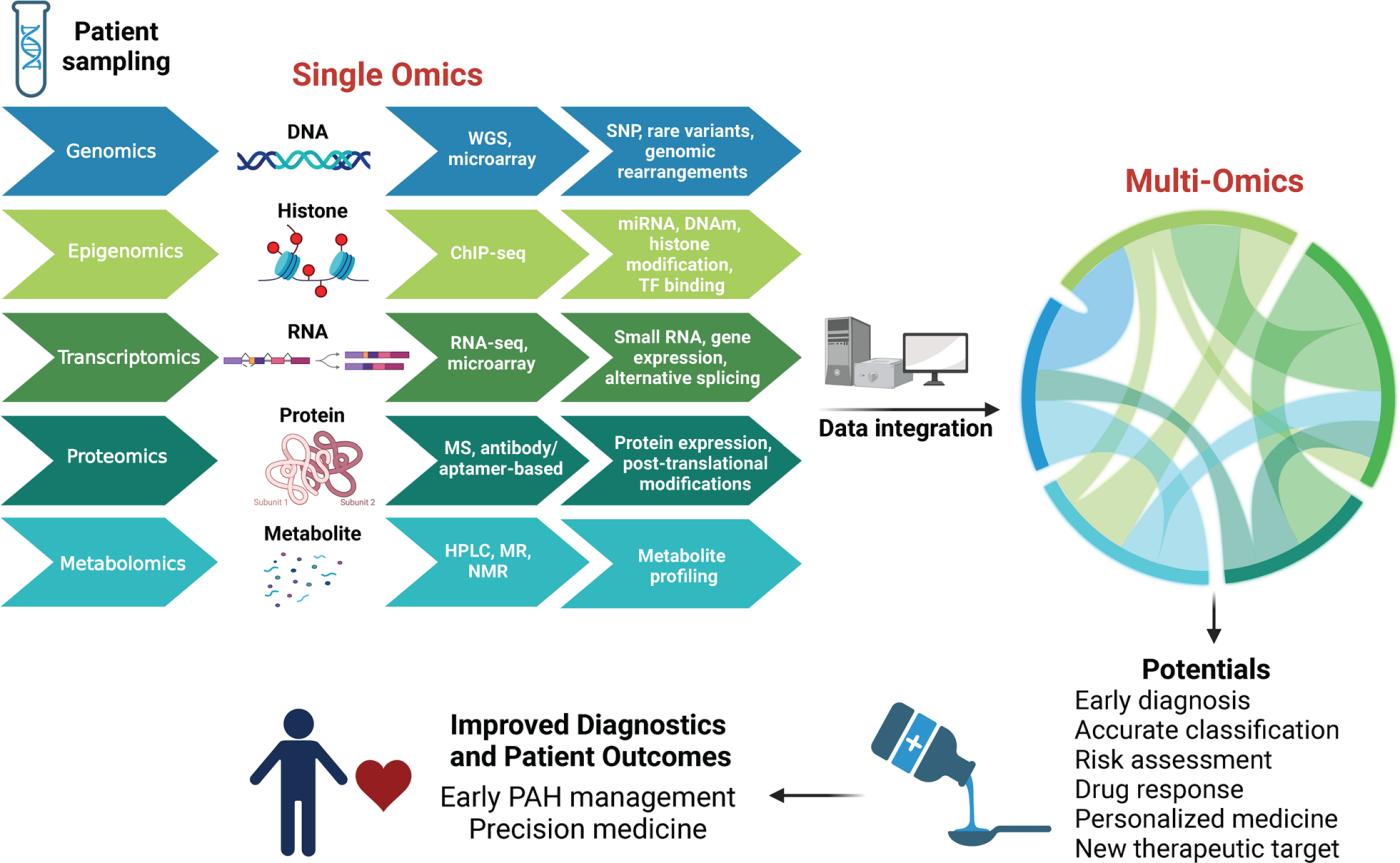
Overview of the relationship between single and multi-omics. Results and data from single omics studies can be integrated into multi-Omics to provide a more comprehensive understanding of genetic changes and associated modifications in complex diseases such as PAH. Through multi-omics, there is an enhanced potential for earlier diagnosis, better treatment efficacy, and a more personalized therapy, resulting in improved patient outcomes. *WGS* whole genome sequencing, *ChIP-seq* chromatin immunoprecipitation sequencing, *RNA-seq* RNA sequencing, *MS* mass spectrometry, *HPLC* high performance liquid chromatography, *MR* mendelian randomization, *NMR* nuclear magnetic resonance, *SNP* single nucleotide polymorphism, *miRNA* micro-RNA, *DNAm* DNA methylation, *TF* transcription factor. Created with BioRender.com

Multi-omics encompasses a range of studies, including, but not limited to, genomics, epigenomics, transcriptomics, metabolomics, proteomics, metagenomics, and pharmacogenomics **(**Fig. 4**)**. Multi-omics analyzes data and provides a more comprehensive picture of the molecular and cellular processes associated with the disease while providing important insight into the best treatment options in a personalized manner. Genomics and metagenomics study the arrangement of genes in an organism to identify genomic variants associated with diseases [130, 131]. Epigenomics then analyzes any changes in the genome, such as DNA methylation, post-transcriptional changes, or post-translational modifications of histones [131]. Transcriptomics examines RNA expression and delves deeper into specific cellular gene expression patterns [131, 132]. Metabolomics goes one step further and provides an idea of the metabolic state of the cell or tissue, disruptions in metabolic pathways such as glycolysis, and its molecular phenome [131, 132]. Proteomics provides a holistic understanding of the functional changes associated with differences in protein expression, abundance, and post-translational modifications [132]. This helps build a bridge between gene expression and subsequent protein expression. Lastly, pharmacogenomics investigates how genes affect responsiveness to drug treatments [131, 132]. Integrating and combining large multi-omics datasets provides a detailed blueprint of an individual’s molecular signature. More precisely, this comprehensive approach enables a better understanding of genetic composition, associated modifications, and expression patterns while facilitating the identification of therapeutic strategies that are more viable and efficacious, ultimately leading to improved outcomes.

As multi-omics data analysis defines the molecular profiles of patients, it can potentially improve the current classification of PAH beyond genetic studies and single-gene mutation tests [129, 133]. Importantly, these data can be combined to better understand PAH, uncover and characterize cellular pathways and processes contributing to disease progression and severity, and identify new therapeutic candidates that can be pharmacologically targeted [130]. Omic technologies have also been used to identify new biomarkers that indicate responses to therapies and drugs. Occasionally, this marker can define the disease state and inform responsiveness to treatment. Risk-variant genes can be isolated using genome-wide association studies (GWAS), thus refining the risk-variant characterization [133]. GWAS data plays a critical role in identifying loci associated with complicated diseases and risk factors associated with such genes [133]. The integration of GWAS data with datasets using functional genomics techniques revealed that some variants were found and enriched within regulatory sequences. This highlights the value of using multi-omics with other publicly available datasets to elaborate on the links between genetic risk factors and disease.

However, there are certain limitations to multi-omics that must be overcome to promote its use in disease diagnosis. The short read-base sequences in sequencing technologies currently prevent the thorough study of disease associations with genotypes. However, this can be resolved by long-read sequencing. The large datasets that need to be analyzed also make multi-omics data integration an intensive undertaking. As data are generated using several platforms, formatting differs and must be modified and processed individually [134]. Another limitation during sample measurement is the lack of data on biomolecules because they may not all be assessed, possibly because of cost, instrument sensitivity, or other experimental factors [135]. However, with recent artificial intelligence developments and publicly accessible datasets, multi-omics data analysis has greatly improved, assuming access to all observed data following sample testing [131, 135].

Overall, the integration of data from multiple omics technologies, such as genomics, transcriptomics, proteomics, and metabolomics, can provide new insights into the disease [37, 136–138] and help identify new molecular biomarkers, and better define the molecular signatures of individual patients, allowing for more personalized treatment approaches [139]. Consequently, multi-omics integration is particularly relevant for complex and chronic diseases, such as cardiovascular disease and cancer, which are influenced by multiple environmental and lifestyle factors [129, 140].

## Conclusion

Epigenetic therapies such as DNA methyltransferase inhibitors, histone deacetylase inhibitors, EZH2, and BET inhibitors have shown promising results in preclinical models of PAH by improving pulmonary hemodynamics, reducing vascular remodeling, and increasing survival. Clinical trials using epigenetic therapies in patients with PAH are currently ongoing, and early results are encouraging. Furthermore, recent studies have highlighted the potential and clinical relevance of multi-omics integration to improve our understanding of the molecular profiles of patients with PAH. These studies are crucial for identifying new therapeutic targets, improving treatment efficacy, and facilitating the development of personalized treatment approaches. However, further research is necessary to better understand the long-term safety and efficacy of these therapies before they can be widely adopted in clinical practice. Overall, integrating multi-omics data with the efficacy of epigenetic therapies is promising and can have a transformative impact on healthcare.

## Data Availability

Not applicable to this article, as no data were generated or analyzed.

## Abbreviations

5-caC: 5-carboxycytosine
5-fC: 5-formylcytosine
5-hmC: 5-hydroxymethylcytosine
5-hmU: 5-hydroxymethyluracil
5-mc: 5-methylcytosine
AAV1: Adeno-associated virus serotype 1
AID/APOBEC: Activation-induced cytidine deaminase/apolipoprotein B mRNA-editing enzyme complex
BMPR2: Bone morphogenetic protein receptor type 2
BRD4: Bromodomain-containing protein 4
CO: Cardiac output
CTGF: Connective tissue growth factor
DNA: Deoxyribonucleic Acid
DNMT: DNA methyltransferase
EPZ-6438: Tazemetostat
EZH2: Enhancer of zeste homolog 2
FHR: Fawn-hooded rats
FPAH: Familial pulmonary arterial hypertension
H3K27me3: Trimethylation of lysine 27 on histone H3
HDAC: Histone deacetylases
HIF-1α: Hypoxia-inducible factor
HPAH: Heritable pulmonary arterial hypertension
IPAH: Idiopathic PAH
KO: Knockout
Kv1.5: Oxygen-sensitive voltage-gated K+ channels
lncRNA: Long non-coding RNA
LSH: Lymphocyte-specific helicases
MCT: Monocrotaline
MeCP: Methyl CpG binding proteins
miRNA/miR: Micro RNA
mPAP: Mean PA pressure
MRI: Magnetic resonance imaging
NHL: Non-hodgkin lymphoma
PA: Pulmonary artery
PAAT: PA acceleration time
PAB: Pulmonary artery banding
PAEC: Pulmonary artery endothelial cells
PAH: Pulmonary arterial hypertension
PASMC: Pulmonary artery smooth muscle cells
PDK: Pyruvate dehydrogenase kinase
PH: Pulmonary hypertension
PPHN: Persistent PH of the newborn
PRC2: Polycomb repressive complex 2
PVR: Pulmonary vascular resistance
REVEAL: Registry to Evaluate Early and Long-term PAH Disease Management
RFTS: Replication foci targeting sequence
RISC: RNA-induced silencing complex
RV: Right ventricle/ventricular
RVfib: Right ventricular fibroblast
RVSP: RV systolic pressure
SAHA: Suberoyl anilide hydroxamic acid
SAM: S-adenyl methionine
SIN3A: Switch-independent 3A
SOD2: Superoxide dismutase 2
SRA: SET and RING RING-associated
Su/Hx: Sugen/Hypoxia
TDG: Thymine-DNA-glycosylase
TET: Ten-eleven translocation enzymes
TGF-β1: Transforming growth factor beta
TPR: Total pulmonary vascular resistance
TRV: Tricuspid regurgitation velocity
TTD-PHD: Tandem tudor domain and plant homeo domain
TTE: Transthoracic echocardiography
TubA: Tubastatin A
UBL: Ubiquitin-like
VPA: Valproic acid
VTI: Velocity time integral
